# Goal directed fluid therapy for major liver resection: A multicentre randomized controlled trial

**DOI:** 10.1016/j.amsu.2019.07.003

**Published:** 2019-07-10

**Authors:** Laurence Weinberg, Damian Ianno, Leonid Churilov, Steven Mcguigan, Lois Mackley, Jonathan Banting, Shi Hong Shen, Bernhard Riedel, Mehrdad Nikfarjam, Chris Christophi

**Affiliations:** a Director of Anesthesia, Austin Hospital; and A/Professor, Department of Surgery, Austin Health, The University of Melbourne, Victoria, Australia; b Department of Anesthesia, Austin Health, Victoria, Australia; c Statistics and Decision Analysis Academic Platform, The Florey Institute of Neuroscience and Mental Health, Melbourne Brain Centre, Victoria, Australia; d Department of Anesthesia, Austin Hospital, Heidelberg, Victoria, Australia; e Department of Anesthesia, Peter MacCallum Cancer Hospital, Victoria, Australia; f Department of Surgery, Austin Hospital, The University of Melbourne, Victoria, Australia; g Department of Medicine (Austin Health), Melbourne Medical School, The University of Melbourne, Victoria, Australia

**Keywords:** Hepatectomy, Surgery, Monitoring, Fluid therapy, Hemodynamics, Cardiac output, Complications

## Abstract

**Background:**

The effect a restrictive goal directed therapy (GDT) fluid protocol combined with an enhanced recovery after surgery (ERAS) programme on hospital stay for patients undergoing major liver resection is unknown.

**Methods:**

We conducted a multicentre randomized controlled pilot trial evaluating whether a patient-specific, surgery-specific intraoperative restrictive fluid optimization algorithm would improve duration of hospital stay and reduce perioperative fluid related complications.

**Results:**

Forty-eight participants were enrolled. The median (IQR) length of hospital stay was 7.0 days (7.0:8.0) days in the restrictive fluid optimization algorithm group (Restrict group) vs. 8.0 days (6.0:10.0) in the conventional care group (Conventional group) (Incidence rate ratio 0.85; 95% Confidence Interval 0.71:1.1; p = 0.17). No statistically significant difference in expected number of complications per patient between groups was identified (IRR 0.85; 95%CI: 0.45–1.60; p = 0.60). Patients in the Restrict group had lower intraoperative fluid balances: 808 mL (571:1565) vs. 1345 mL (900:1983) (p = 0.04) and received a lower volume of fluid per kg/hour intraoperatively: 4.3 mL/kg/hr (2.6:5.8) vs. 6.0 mL/kg/hr (4.2:7.6); p = 0.03. No significant differences in the proportion of patients who received vasoactive drugs intraoperatively (p = 0.56) was observed.

**Conclusion:**

In high-volume hepatobiliary surgical units, the addition of a fluid restrictive intraoperative cardiac output-guided algorithm, combined with a standard ERAS protocol did not significantly reduce length of hospital stay or fluid related complications. Our findings are hypothesis-generating and a larger confirmatory study may be justified.

## Highlights

• Major liver resection is a complex procedure with up to 40% patients experiencing complications.
• Optimisation of perfusion and oxygen delivery to all organs remain the cornerstone of best hemodynamic care.
• Traditionally, a low central venous pressure strategy during major liver resection has been used to reduce venous bleeding.
• The impact of a restrictive cardiac output fluid optimisation algorithm during major liver surgery is unknown.
• After major hepatobiliary surgery, a fluid restrictive algorithm did not reduce length of hospital stay or complications.

## 1 Introduction

Major liver resection remains a complex procedure with up to 40% patients experiencing complications, even in high volume centres [1]. Optimization of perfusion and oxygen delivery to the residual liver and other organs, whilst avoiding hyper and hypovolemia, remain the cornerstones of best hemodynamic care. This, in addition to contemporary Enhanced Recovery After Surgery (ERAS) programmes for liver resection is associated with reduced intraoperative bleeding, perioperative complications and hospital length of stay [[2], [3], [4], [5], [6], [7]]. Traditionally, fluid intervention for major hepatic resection includes fluid restriction and low central venous pressure during the dissection and transection phases to reduce venous bleeding, with restoration of euvolemia post transection with judicious fluid intervention. Recently, in a large pragmatic multicentre trial of 3000 patients at increased risk for complications during major abdominal surgery (RELIEF trial) [8], a restrictive fluid regimen was not associated with a higher rate of disability-free survival than a liberal fluid regimen and was associated with a higher rate of acute kidney injury. Although this study is the largest trial to date exploring restrictive and liberal fluid strategies, the study excluded patients undergoing liver resection surgery. The impact of a restrictive cardiac output fluid optimization algorithm in addition to an established liver ERAS protocol is unknown. Therefore, we hypothesized that for patients undergoing major hepatic resection with an ERAS protocol, the addition of a restrictive intraoperative fluid optimization algorithm will improve duration of hospital stay and reduce perioperative fluid related complications.

## 2 Materials and methods

After Research and Ethics Committee approval (no: 05006/2013) we conducted a multicentre, randomized study with a two-arm parallel group design at two university hospitals and one private hospital, each with a high volume and dedicated hepatobiliary service. All patients provided their written informed consent for participation in the research study. The study was conducted between September 2013 and May 2016 and registered with an international clinical trials registry on 11/02/2016 (Australian New Zealand Clinical Trials Registry number: 12616000172404). There were no changes to the original study protocol endpoints (primary or secondary) approved by Research and Ethics Committee, and participant consent and randomization began after institutional ethics approval was obtained. (Key trial dates: trial protocol finalized: 23/04/2013; submitted for institutional ethics approval: 23/04/13; research ethics committee approval date: 03/09/2013; first participant enrolled: 09/09/2013; last participant enrolled: 23/05/2016; project completion: 01/06/2016).

A team of 6 expert hepatobiliary anesthesiologists and surgeons working across all sites performed the cases. The ERAS protocol was identical in all institutions allowing for complete homogeneity in perioperative care as it pertains to major liver resection (Supplementary Table 1). All anesthesiologists were expertly trained in the use of the FloTrac sensor EV1000 hemodynamic platform.

### 2.1 Eligibility criteria

Participants were identified from pre-assessment clinics and included adult patients (greater than 18 years) undergoing elective major liver resection. We defined major resection as a resection of 3 or more liver segments. The following patients were excluded: preoperative coagulopathy (international normalized ratio >1.5), thrombocytopenia (platelet count <75 × 10^9^/L), renal impairment (creatinine >250* μ*mol/L), hepatic insufficiency (bilirubin >30* μ*mol/L, albumin <25 g/dL, alkaline phosphatase >300U/L, alanine transaminase >50U/L), American Society Anesthesiology class > III, impaired left ventricular function (ejection fraction <40%), atrial fibrillation, moderate or severe tricuspid regurgitation or any impairment of right ventricular function.

### 2.2 Randomization and blinding

An independent statistician generated a computerised sequence of 50 allocation codes, 25 for each group using commercial software (www.randomization.com). An independent research nurse sealed the allocation codes into sequentially numbered opaque envelopes. Randomization was done by the chief investigator from a central unit on the day of surgery and prior to induction of anesthesia. Statistics were done by a statistician blinded to allocation and the code was broken after analysis was completed. The study participants, surgeons, and all staff involved in postoperative care were blinded to treatment assignments. Participants randomized to conventional care had the advanced hemodynamic display screen of the EV100 monitor covered with an opaque screen. All alarms were deactivated.

### 2.3 Intervention

**Fig. 1:**
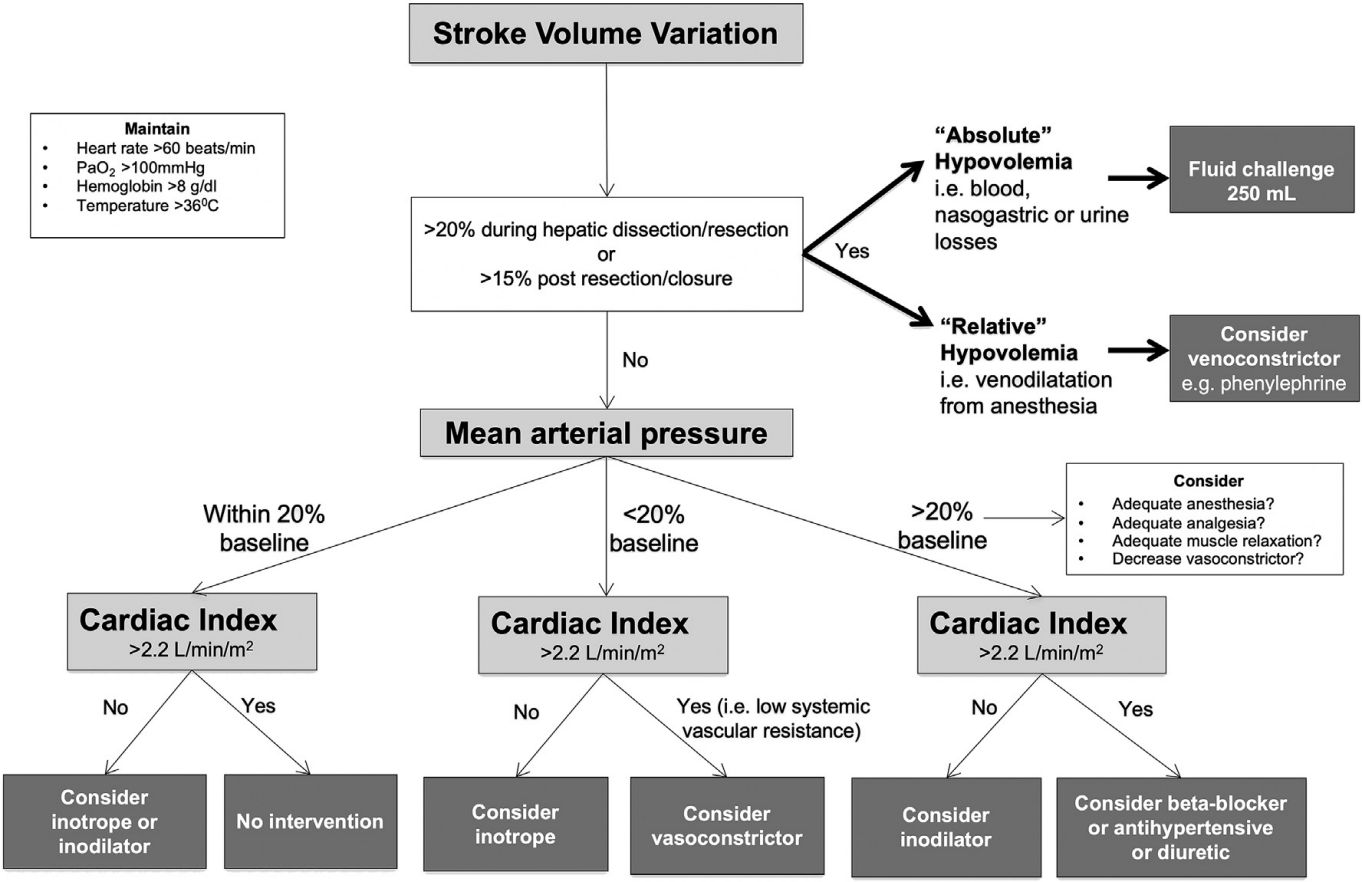
Fluid restrictive cardiac output algorithm for patients undergoing major liver resection.

For participants in the Restrict group, fluid intervention and delivery of vasoactive therapies were directed by a physiologic fluid cardiac output optimization algorithm based on physiological parameters from the EV1000 hemodynamic monitor (Fig. 1). A SVV of greater than 20% was used as a threshold for fluid intervention during the dissection and hepatic transection stages. During hemostasis and surgical closure a SVV target of greater than 15% was used as a fluid intervention target for restoration of euvolemia. For participants in the Conventional care group, fluid therapy and use of vasoactive therapy was at the discretion of the anesthesiologist. Generally, according to standard anesthesia practices at all institutions, this entailed maintenance of low CVP (less than 8 mmHg) during the pre-hepatic transection and dissection phases. Fluid restriction and reverse Trendelenburg positioning were employed if necessary, to assist in low central venous pressure anesthesia. Similarly, intravenous glyceryl trinitrate (5–20 μg/min) was used to further reduce central venous pressure if clinically required. After completion of the liver transection, euvolemia was restored with judicious fluid intervention that consisted of a balanced crystalloid, 4% or 20% albumin solution, or blood/blood products if clinically indicated.

### 2.4 Clinical pathway for all participants

Preoperatively, all participants underwent a comprehensive multidisciplinary assessment, with optimization of hemoglobin [9], nutrition and medical comorbidities. All participants underwent a standardized ERAS programme outlined in Supplementary File 1. On arrival to the operating room all participants had intravenous access established and an arterial line inserted into the radial artery of the non-dominant hand. A FloTrac™ catheter (FloTrac System 4.0, Edwards Lifesciences, Irvine, CA, USA) was then attached to the participant's arterial line that was then connected to an EV1000 hemodynamic monitor (Edwards Lifesciences, Irvine, CA, USA), which provided real time measurements of continuous blood pressure, cardiac and stroke volume index, stroke volume variation (SVV), and systemic vascular resistance. The arterial transducer was zeroed to atmospheric pressure at the level of the right atrium. Acute normovolemic hemodilution was not utilized in any of the institutions. All vasoactive medications were administered via a central venous catheter. All patients were mechanically ventilated with 8 mL/kg tidal volume and 5 cmH_2_O of positive end expiratory pressure. Blood pressure was defended to within 20% of the patient's baseline value.

### 2.5 Primary and secondary outcomes

The primary outcome was duration of hospital stay, which was measured from the time surgery was completed (last suture) to hospital discharge. Discharge criteria included the following parameters: i) unassisted mobilization ii) restoration of oral and solid intake iii) satisfactory analgesia, and iv) the absence of, or recovery from any perioperative complication. Secondary outcomes included the following: i) expected number of complications per patient ii) number (proportion) of participants with at least 1 complication iii) volume of intraoperative fluid use (expressed as a total volume, and as millilitres per kilogram per hour); iv) intraoperative fluid balances; v) use of intraoperative vasoactive medications. Exploratory outcomes included intraoperative hemodynamic differences between the groups recorded EV1000 hemodynamic monitor. Intraoperative third space losses were considered negligible. Perioperative fluid balance was calculated by subtracting total fluid output (e.g. blood and urine) from total fluid input (inclusive of all parenteral and enteral intake).

We defined complications as unexpected deviations from standard care using the European Perioperative Clinical Outcome (EPCO) definitions [10]. Bile leak was defined as presence of bile in the drainage fluid that persisted on postoperative day 4, and acute pancreatitis was defined as an elevation in serum lipase greater than three times the normal laboratory range. All complications were reviewed by two authors independently and graded according to the Clavien-Dindo Classification [11]. Any discrepancy was resolved by an independent clinician.

### 2.6 Sample size

We used our institution's liver resection outcome database for sample size calculations. We used inference for means comparing two independent samples with a two-tailed *t*-test. With a mean length of hospital stay of 6.2 days and a standard deviation of 1.5 days, assuming a two-tailed threshold for statistical significance of 0.05, we estimated a total sample size of 50 participants (equally distributed between two arms) will yield an 80% power to observe a large treatment effect (Cohen's d = 0.8 or higher, corresponding to at least a one-day difference) between the Conventional care and Restrict group.

### 2.7 Statistics

We used commercial statistical software (STATA/IC v.13. and Prism 7.0 GraphPad (La Jolla, CA, USA). Variables were summarized as either a median (interquartile range, IQR) and compared using Mann-Whitney *U* test, or as counts (proportions) and compared using Chi-square or Fisher's exact test as appropriate. To investigate the effects of GDT on individual outcomes we used applicable regression models. For length of hospital stay (treated as the count of days) and for per patient count of postoperative complications we used negative binomial regression models. Fluid outcomes were measured with linear regression models with robust standard error estimation and use of individual vasoactive drugs were measured with logistic regression models. Corresponding effects are summarized as either Incidence Rate Ratios (IRRs), Odds Ratios (ORs) or mean differences with corresponding 95% confidence intervals (CI). The difference between groups in distributions of highest per-patient grade of complications was estimated using Wilcoxon-Mann-Whitney Generalized odds ratio and corresponding 95% CI. A two-tailed p value of 0.05 was chosen as the threshold to indicate statistical significance. We followed the CONSORT guidelines for reporting randomized trials [12]. No interim analysis was conducted.

## 3 Results

**Fig. 2:**
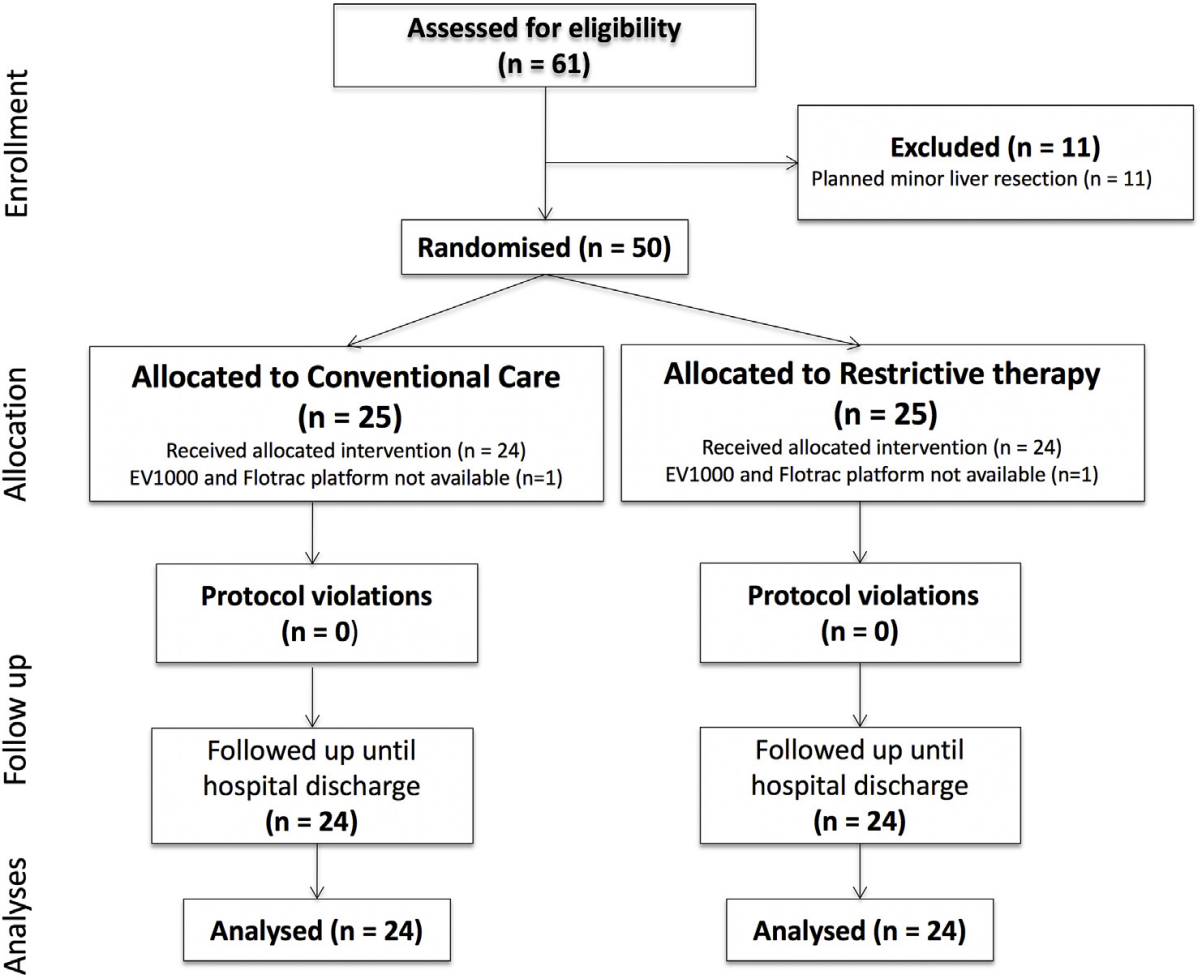
Consort diagram.

Sixty-one patients were screened for eligibility. Eleven patients were excluded who underwent planned minor liver resection surgery. Remaining participants were consented; twenty-five participants were randomized to the restrictive fluid optimization algorithm group (Restrict group) and twenty-five to conventional care (Conventional group) (Fig. 2). One subject was excluded from each group as the EV1000 Platform and Flotrac catheter were not available on the day of surgery. This was considered as missing completely at random. There were no violations or breaches of the study or ERAS protocols. There was no unintentional unblinding. There was no harm or unintended effect of the intervention in either group. Baseline participant characteristics are presented in Table 1. The median (IQR) age was 64 years (57:71) in the Restrict group and 61 years (52:73) in the Conventional care group respectively.

**Table 1:** Characteristics of patients undergoing major liver resection with and without restrictive fluid therapy. Data presented as median (interquartile range) or number (proportion).

	Restrict group (n = 24)	Conventional care group (n = 24)
**Characteristics**		
Age (years)	64 (57:71)	61 (52:73)
Male:Female	17:7	15:9
Body mass index (kg/m^2^)	27 (24:33)	27 (23:31)
ASA Class I-II	6 (25%)	3 (13%)
ASA Class III	18 (75%)	21 (88%)
Diabetes	5 (21%)	3 (13%)
Dyslipidemia	7 (29%)	4 (17%)
Chronic obstructive pulmonary disease	1 (4%)	1 (4%)
Hypertension	8 (33%)	12 (50%)
Ischemic heart disease	5 (21%)	3 (12%)
Peripheral vascular disease	3 (13%)	3 (12%)
Malignancy	23 (96%)	23 (96%)
**Preoperative bloods**		
Hemoglobin (g/L)	137 (129:152)	136 (125:148)
White cell count (x10^∧9^/L)	6.1 (5.2:7.2)	6.6 (4.6:8.0)
Platelets (x10^∧9^/L)	211 (172:265)	208 (161:267)
Albumin (g/L)	42 (37:43)	41 (39:44)
Bilirubin (μmol/L)	8 (6:11)	8 (7:11)
Creatinine (μmol/L)	75 (59:86)	76 (66:98)
eGFR (mL/min/1.73m^2^)	88 (76:90)	82 (68:90)
Alkaline phosphatase (U/L)	92 (71:122)	124 (96:163)
Alanine aminotransferase (U/L)	33 (20:62)	32 (19:50)
International normalized ratio	1 (1:1.1)	1 (1:1.1)
Prothrombin time (seconds)	11.6 (11.0:12.4)	11.5 (11.0:12.0)
Activated partial thromboplastin time (seconds)	25.5 (24.3:27.0)	27.0 (25.8:32.3)

ASA – American society of anesthesiologists.

**Fig. 3:**
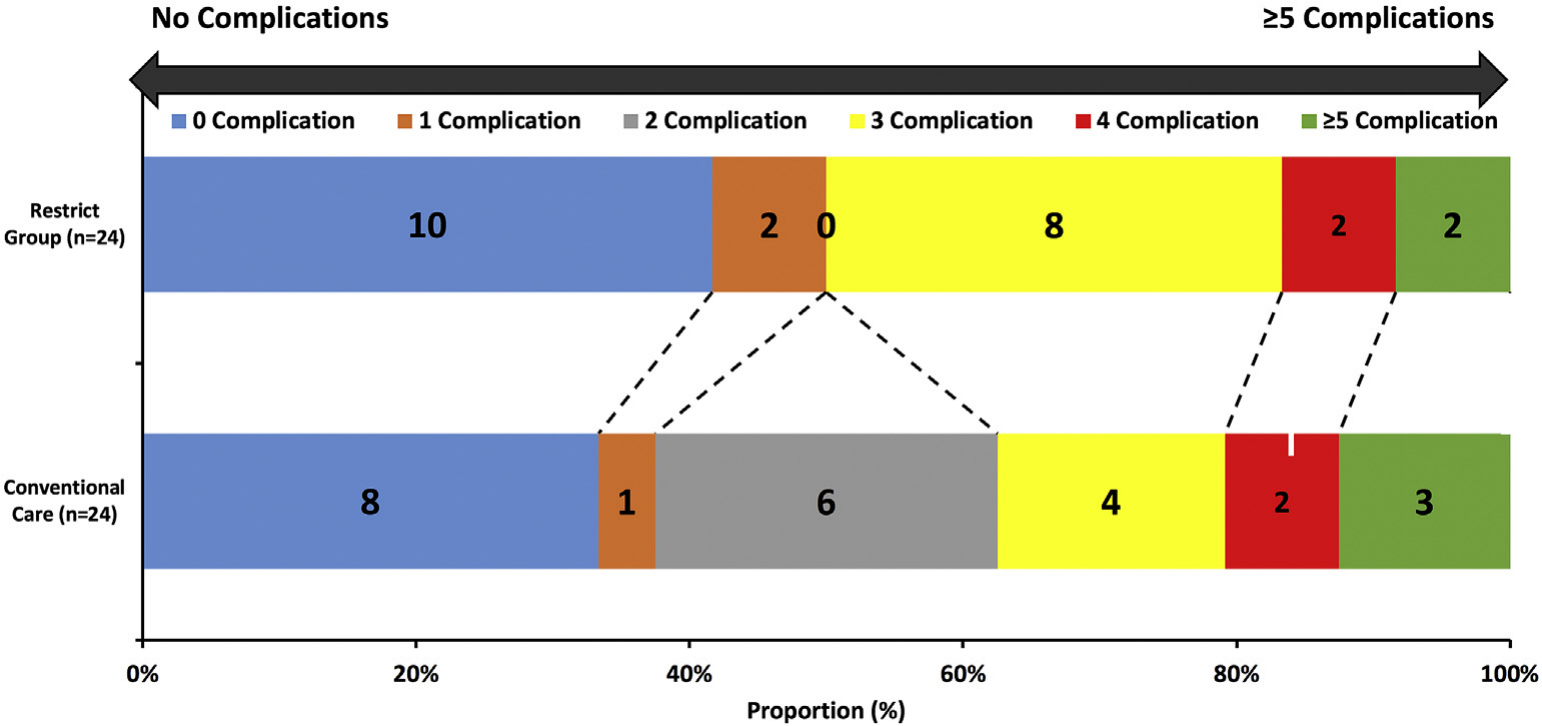
The distributions of participants across categories defined by number of complications per participant in the restrictive fluid therapy group (Restrict group) and Conventional care group.

For the primary end point, the median (IQR) length of stay was 7.0 days (6.0:8.0) in the Restrict group vs. 8.0 days (6.0:10.0) in the Conventional care group [Incidence rate ratio 0.85; 95%CI 0.71:1.1; p = 0.17]. No statistically significant differences in expected number of complications per patient between group was identified (IRR 0.85; 95%CI: 0.45–1.60; p = 0.60). Fourteen participants (58%) developed a complication in the Restrict group compared to 16 (67%) in the Conventional care group (p = 0.55). The number of complications per participant in the Restrict group and Conventional care groups is presented graphically in Fig. 3. Most complications were Clavien-Dindo grade I and II (Table 2). The generalized odds of having a worse complication for a random participant in the Restrict group compared to a random participant in the Conventional care group were 0.79 (95%CI: 0.27–1.96; p = 0.71). Nine participants (37.5%) in the Restrict group developed cardiorespiratory complications vs. 13 (54%) in the Conventional care group (p = 0.25). The incidence of acute kidney injury [four participants (17%) for both groups] and requirement for blood transfusion [four participants (17%) in the Restrict group Restrict group and three participants (13%) for Conventional care group] was similar. The highest median (IQR) postoperative creatinine in the Restrict group was 79 μmol/L (69.5:90.2) vs. 90 μmol/L (73.0:124) in the Conventional care group (p = 0.15). The lowest median postoperative estimated glomerular filtration rate (eGFR) in the Restrict group was 87.5 mL/min/1.73 m^2^ (75.5:90.0) vs. 82 mL/min/1.73 m^2^ (68:90) in the Conventional care group (p = 0.15). The median (IQR) length of intensive care stay was similar: 11.5 h (9:17) in the Restrict group vs. 11.0 h (9:15) in the Conventional care group (p = 0.63). Detailed intraoperative hemodynamic data between the groups are presented graphically in the Supplementary File Figs. 1–7.

**Table 2:** Postoperative complications. Data presented as number (proportion). Effect size reported as odds ratio (95%Confidence Interval).

	Restrict group (n = 24)	Conventional care group (n = 24)	Effect size (95%CI)	p value
Length of hospital stay (days)	7.0 (6.0:8.0)	8.0 (6.0:10.0)	0.86 (0.71:1.1)^a^	0.17
Patients with a complication	14 (58%)	16 (67%)	0.70 (0.22–2.26)^b^	0.55
Number of complications per patient	1.8 (1.8)	2.2 (2.1)	0.85 (0.45:1.59)^a^	0.60
Clavien Dindo Grade of most severe complication			0.79 (0.27–1.96)^c^	0.71
Grade I	3 (13%)	3 (13%)		
Grade II	10 (42%)	11 (46%)		
Grade III	1 (4%)	1 (4%)	Not estimable^b^	
Grade IV	0	0		
Grade V	0	1 (4%)		
Wound infection	3 (13%)	5 (21%)	0.54 (0.11:2.58)	0.44
Superficial surgical site infection	3 (13%)	4 (17%)	0.71 (0.14:3.60)	0.68
Deep surgical site infection	0	1 (4%)	Not estimable	>0.99
Delayed gastric emptying	2 (8%)	2 (8%)	1 (0.13:7.75)	>0.99
Bile leak	1 (4%)	2 (8%)	0.48 (0.04:5.66)	0.56
Cardiorespiratory complications	9 (37.5%)	13 (54%)	0.51 (0.16:1.61)	0.25
Acute respiratory distress syndrome	1 (4%)	1 (4%)	1 (0.06:16.97)	>0.99
Pneumonia	1 (4%)	2 (8%)	0.48 (0.04:5.66)	0.56
Pulmonary atelectasis	4 (17%)	3 (13%)	1.40 (0.28:7.06)	0.68
Pulmonary congestion	0	2 (8%)	0.40 (0:5.29)	0.49
Cardiogenic pulmonary oedema	1 (4%)	1 (4%)	1 (0.06:16.97)	>0.99
Arrhythmia	2 (8%)	4 (17%)	0.45 (0.07:2.76)	0.39
Acute pancreatitis	1 (4%)	1 (4%)	1 (0.06:16.97)	>0.99
Acute kidney injury	4 (17%)	4 (17%)	1 (0.22:4.56)	>0.99
Delirium	1 (4%)	3 (13%)	0.30 (0.03:3.16)	0.32
Liver failure	0	1 (4%)	Not estimable	>0.99
Nausea and vomiting	2 (8%)	1 (4%)	2.09 (0.18:24.73)	0.56
Electrolyte disturbances	11 (27%)	8 (62%)	1.69 (0.53:5.44)	0.377
Hypokalemia	7 (29%)	3 (13%)	2.88 (0.65:12.87)	0.165
Hypomagnesemia	2 (8%)	2 (8%)	1 (0.13:7.75)	>0.99
Hypophosphatemia	2 (8%)	3 (13%)	0.64 (0.10:4.20)	0.64
Endocrine abnormalities	3 (13%)	1 (4%)	3.29 (0.32:34.08)	0.32
Drug reaction	1 (4%)	0	Not estimable	>0.99
Refractory analgesia	0	1 (4%)	Not estimable	>0.99
Other^c^	2 (8%)	3 (13%)	0.64 (0.10:4.20)	0.64
Required blood transfusion	4 (17%)	3 (13%)	1.4 (0.28:7.06)	0.68
Return to theatre	1 (4%)	2 (8%)	0.48 (0.04:5.66)	0.56
Unplanned intensive care unit admission	0	1 (4%)	Not estimable	>0.99

^d^Average difference with robust 95%CI.

a Incidence rate ratio.

b Odds ratio.

c Generalized odds ratio.

Median (IQR) duration of surgery was 5.5 h (3.3:8.0) in the Restrict group vs. 4.8 h (4.0:5.5) in the Conventional care group (p = 0.15). Total median (IQR) intraoperative fluid use was 2000 mL (1050:2449) in the Restrict group and 2000 mL (1500:2875) in the Conventional care group (p = 0.18). The median fluid infusion rate was 4.3 mL/kg/hr (2.6:5.8) in the Restrict group vs. 6.0 mL/kg/hr (4.2:7.6) in the Conventional care group (p = 0.03). No significant differences in the total volumes of crystalloid, colloid or blood products administered were observed (Table 3). Fluid balance was lower in the Restrict group: 808 mL (571:1565) vs. 1345 mL (900:1983) in the Conventional care group; p = 0.05. Four patients (17%) in the Restrict group received intraoperative metaraminol vs. 11 patients (46%) in the Conventional care group (p = 0.06). Three participants (13%) received intraoperative ephedrine in the Restrict group vs. 6 (25%) in the Conventional care group (p = 0.46). Seventeen participants (71%) in the Restrict group received intraoperative noradrenaline or phenylephrine vs. 13 (54%) in the Conventional care group (p = 0.37). The use of dopamine and/or dobutamine was not significantly higher in the Restrict group compared to the Conventional care group: 7 participants (29%) vs. 3 (13%) (p = 0.29). Postoperative fluid intervention is presented in Table 4.

**Table 3:** Intraoperative fluids and vasoactive medications in undergoing major liver resection with and without restrictive fluid therapy. Data presented as number (proportion). Effect size reported as odds ratio (95% Confidence Interval).

	Restrict group (n = 24)	Conventional care group (n = 24)	Effect size (CI)	p value
Total fluid intraoperatively (mL) (including colloids, crystalloids and blood)	2000 (1050:2449)	2000 (1500:2875)	−388 (−958:183)^a^	0.18
Median fluid infusion rate (mL/kg/hr)	4.3 (2.6:5.8)	6.0 (4.2:7.6)	1.69 (0.13: 3.25)^a^	0.03
Total fluid balance (mL)	808 (571:1565)	1345 (900:1983)	537 (26:1071)^a^	0.05
Urine output (mL)	290 (163:563)	280 (173:304)	−131.5 (−276:12.9)^a^	0.07
**Crystalloid therapy**				
No of patients	24 (100%)	24 (100%)		>0.99
Total volume (mL)	1875 (1000:2000)	2000 (1125:2000)		0.25
**Colloid therapy (excluding blood)**				
No of patients	15 (62.5%)			>0.99
Total volume (mL)	200 (500:700)	14 (58%)		0.44
*20% albumen*		200 (175:550)		
No of patients	7 (29%)			>0.99
Total volume (mL)	200 (200:200)	8 (33%)		>0.99
*4% albumen*		200 (125:200)		
No of patients	6 (25%)			0.75
Total volume (mL)	500 (500:1000)	8 (33%) 500 (500:875)		0.66
				
**Blood transfusion**				
No of patients	2 (8%)			
Total volume (mL)	356 (248:465)	1 (4%)		>0.99
		465 (465:465)		Not estimable
**Any vasoactive/inotrope**	23 (96%)	22 (92%)	2.09 (0.18:24.7)^b^	0.55
Metaraminol	4 (25%)	11 (46%)		0.06
Norepinephrine or phenylephrine	17 (71%)	13 (53%)		0.37
Ephedrine	3 (12.5%)	6 (25%)		0.46
Dopamine/dobutamine	7 (29%)	3 (12.5%)		0.29
Metoprolol/esmolol	1 (4%)	2 (8%)		>0.99

a Incidence rate ratio.

b Odds ratio.

**Table 4:** Postoperative fluid intervention in patients undergoing major liver resection with and without restrictive fluid therapy. Data presented as number (proportion) and median (interquartile range). Effect size reported as odds ratio (95% Confidence Interval).

	Restrict group (n = 24)	Conventional care group (n = 24)	Effect size (95%CI)	p value
**Day 1**				
Crystalloids (mL)	2534 (2082:3052)	1947 (1534:2615)	618 (51:1184)^a^	0.03
Colloids (mL)	0 (0:438)	0 (0:500)	−78 (−296:140)^a^	0.48
Blood products	1 (4%)	0	Not estimable^b^	>0.99
Total IV fluid (mL)	2952 (2372:3435)	2259 (1609:2860)	577 (−31:1186)^a^	0.06
Fluid balance (mL)	1535 (757:2238)	1727 (1072:2350)	79 (−494:652)^a^	0.78
Urine output (mL)	1375 (866.3:2275)	1025 (692.5:1550)	−294 (−743:154.9)^a^	0.19
**Day 2**				
Crystalloids (mL)	1346 (760:1710)	1249 (960:1777)	−116 (−562:330)^a^	0.60
Colloids (mL)	0 (0:0)	0 (0:0)	−54 (−160:53)^a^	0.32
Blood products	0	3 (13%)	0.20 (0:1.91)^b^	0.17
Total IV fluid (mL)	1346 (760:1802)	1249 (960:1777)	−235 (−756:289)^a^	0.37
Fluid balance (mL)	648 (−127:775)	593 (−396:1317)	−283 (−968:402)^a^	0.41

Median (IQR) intraoperative urine output was 290 mL (163:563) in the GDT group vs. 280 mL (173:304) in the Conventional care group (p = 0.07). Median (IQR) urine output on postoperative Day 1 was 1375 mL (866.3:2275) in the GDT group vs. 1025 mL (692.5:1550) in the Conventional Care group (p = 0.19).

## 4 Discussion

In this multicentre randomized control trial, the intraoperative application of a fluid restrictive physiological cardiac output algorithm did not result in a significantly shorter duration of hospital stay or lower rates of complications after major liver resection. Participants randomized to the restrictive goal directed therapy fluid protocol combined with an enhanced recovery had lower rates of intraoperative volumes of fluid administered, however there were no significant between-group differences for other individual components of the secondary outcomes.

A unique aspect of the design of this randomized trial was the implementation of a fluid restrictive hemodynamic algorithm using a high SVV. Whilst the beneficial effects of liver resection ERAS protocols in improving length of stay and adverse outcomes are well described [[2], [3], [4], [5], [6], [7]], the use of a fluid restrictive physiological algorithm in patients undergoing major liver resections has not been tested within the framework of a multicentre study. Lin et al. applied a goal directed therapy (GDT) protocol using SVV for resuscitation after low central venous pressure assisted liver resection [3]. Their single centre study differed to ours in several ways. First, both major and minor resections were included. Second, participants were randomized to GDT or conventional care only after the liver transection stage was completed. Finally, Lin et al. employed a fixed fluid dosing regimen with a SVV value ≤ 2 standard deviations from their baseline after induction, whilst we used a SVV value of >20%. This threshold has been reported to be a clear target for fluid responsiveness [13,14]. Similar to our findings, Lin et al. reported that total fluid administration was similar, GDT did not impact on the incidence of postoperative complications and length of hospital stay.

In our study, the median intraoperative infusion rates in the Restrict group (4.3 mL/kg/hr) and Conventional care groups (6.0 mL/kg/hr) were lower than the restrictive (6.5 mL/kg/hr) and liberal (10.9 mL/kg/hr) fluid arms in the RELIEF trial [8]. In contrast to the findings of the restrictive arm in the RELIEF trial, we did not observe a higher incidence of postoperative acute kidney injury or surgical site infection between our groups. However, the incidence of acute kidney injury was 17% in both the Restrict and Conventional care groups of our study, approximately twice as high as the incidence of acute kidney injury reported in the restrictive arm of the RELIEF trial participants. The RELIEF trial did not include patients undergoing liver resection surgery, so the findings of our study may not be generalizable to other types of surgeries.

Traditionally CVP has been utilized as an indicator of intravascular volume status during hepatic resection surgery, and maintenance of a low CVP has been associated with reduced blood loss [15]. More recently however, SVV has been proposed as a minimally invasive and more accurate metric for the dynamic assessment of intravascular volume status. The maintenance of a SVV in the range of 10–20% during major hepatic resection surgery has been shown to correlate with a low CVP [16], and there is growing consensus that targeting a high SVV may be a safe alternative to fluid therapy guided by CVP measurements [16,17]. The SVV target cut off used in our study is aligned with other research groups who report that a CVP between −1 and 1 mmHg strongly correlates to an SVV of 18%–21%; these authors advocate for using a high SVV as a safe alternative to CVP monitoring during hepatic transection [16,17].

The threshold at which SVV should trigger intervention in fluid management during major liver resection remains unclear. Two studies have compared the effect of targeting a high SVV (10–20%) to a low SVV (<10%) during hepatic resection [18,19]. Both reported reduced blood loss in the high SVV group. Both studies utilized a methodology by which the desired range of SVV was achieved using a combination of fixed rates of fluid administration (lower rates in the intervention group) and the additional use of diuretics. Interestingly, the total volume of fluid given in our intervention group appears to be significantly less than that given in the high SVV group in the Seo et al. study [19]. In their study, total fluid dose in mL/kg/hr was not reported. Our study did not show any significant effect of the goal-directed hemodynamic algorithm on blood loss; however, blood loss was not the primary outcome of the study and, similar to other trials, blood loss was based on visual inspection of suction output and assessment of surgical packs, which may be inaccurate and prone to underestimation [20,21]. Both prior studies included only donor transplant patients whilst our study included subjects who required hepatic resection to treat primary hepatobiliary cancer and colorectal metastases.

One other study has examined the use of a goal-directed hemodynamic algorithm to guide fluid resuscitation in the post resection phase of the procedure [1]. Correa-Gallego compared resuscitation guided by SVV variation (bolus fluid administration for an SVV > 2 standard deviations from baseline) to a 6 mL/kg/hr fluid protocol in the conventional care group. Although the intervention was restricted to the post resection phase of the procedure, it was associated with significantly less fluid administration. Similar to our findings, targeting a high SVV to guide fluid therapy showed no significant difference in postoperative complications. We observed a higher complication rate across both groups compared to those reported in a number of large studies of patients undergoing liver resection [22]. However, the majority of complications in our study were Clavien Dindo Grades I and II. A prospective study of morbidity and mortality over 20 years following liver resection utilising the same classification found a rate of complications of 25–39% [23]. The inclusion of only major resections in our study may explain the higher incidence of complications observed.

Our study has several methodological strengths. This was a small multicentre pilot study of adult patients undergoing open major liver resection thereby increasing the external validity of our findings to other centres performing similar surgeries. Our findings may not be applicable to patients undergoing minor resection, laparoscopic resections, or to paediatric patients. However, our sample population shared several unique characteristics in that all anesthesiologists were expert in the both provision of anesthesia for major liver resection surgery and in the use of the advanced hemodynamic platform used, thereby increasing the compliance and adherence to the intervention. The physiological cardiac output hemodynamic algorithm used was pragmatic, allowing the anesthesiologist to administer the therapeutic agents that they were most familiar with as opposed to specified vasoactive medications and fluids. The algorithm considered the patient's baseline physiological state as well as targeting clinically important hemodynamic goals i.e. a patient-specific and surgery-specific individualized approach. Similar to our previous studies in major hepatobiliary-pancreatic surgery [24,25], and in contrast to conservative recommendations, we employed a SVV of 20% as a clearly-defined threshold for the administration of fluid. Stroke volume variation between 9% and 13% has been considered as a questionable threshold for a definitive increase in stroke volume in response to fluid intervention in approximately 25% of patients undergoing surgery [13,14]. Our study utilizes a high SVV threshold for fluid intervention, resulting in a more fluid restrictive protocol than most previous conventional GDT protocols [[26], [27], [28], [29], [30], [31]]. Our findings suggest that this threshold is acceptable for major liver resection surgery during the pre-resection and resection phases of surgery, whilst there was no demonstrable benefit, there was also there was no evidence of harm from this restrictive approach i.e. no observed increased incidence of acute kidney injury, myocardial ischemia etc.

This study has a number of limitations. The vast majority (96% in each group) of patients underwent resection for malignancy although information on pathological diagnosis was not collected. However, the focus of the study was on the association of non-surgical factors with length of hospital stay and the development of complications, regardless of indication for surgery. The outcomes of individual surgeons, anesthesiologists and hospitals were not collected, however all surgeons and anesthesiologists involved in the study work ubiquitously across numerous hepatobiliary facilities. Importantly, the study was powered to identify a reduction in hospital length of stay and a much larger study is likely to be required in order to identify any difference in postoperative complications. This study limited the use of GDT to the intraoperative phase. A previous study utilising GDT in the postoperative phase as part of an ERAS program has shown an association with reduced complications and length of stay [2]. Finally, the 95% confidence intervals reported for length of hospital stay and postoperative complications include clinically relevant values for a potential reduction in these metrics. Therefore, whilst our findings are not statistically significant, there is a suggestion of a clinical effect, and a larger clinical trial may still be justified. Further, corroborative analyses with other similar studies could also be performed to evaluate the consistency and robustness of our findings.

In conclusion, our findings support the feasibility of using a patient-specific, surgery-specific fluid restrictive physiological algorithm to optimize intravenous fluids and vasoactive medications during major liver resection. When combined with an established liver ERAS protocol in high-volume hepatobiliary institutions, a restrictive intraoperative cardiac output-guided algorithm did not significantly reduce length of hospital stay or prevent postoperative complications. The findings from this pilot trial are hypothesis-generating and a larger confirmatory study may be justified.

## Ethics approval

The Austin Health Human Research Ethics Committee approved this study (Approval no: 05006/2013).

This research complied with the principles laid down in the Declaration of Helsinki (Recommendations guiding physicians in biomedical research involving human subjects. Adopted by the 18th World Medical Assembly, Helsinki, Finland, June 1964, amended by the 29th World Medical Assembly, Tokyo, Japan, October 1975, the 35th World Medical Assembly, Venice, Italy, October 1983, and the 41st World Medical Assembly, Hong Kong, September 1989).

All patients provided their written informed consent for participation in the research study. The full trial protocol is available from the corresponding author on request.

## Funding sources

This study was supported by the Department of Anesthesia Research Fund, Austin Health, Victoria, Australia.

## Author contribution

**Laurence Weinberg:** chief investigator, coordination of study at all sites, trial governance, study concept and trial design, recruitment of participants, data analysis and interpretation, writing the paper.

**Damian Ianno:** data collection, formulation of database, data analysis and interpretation, writing the paper.

**Leonid Churilov:** data analysis and interpretation, writing the paper.

**Lois Mackley:** data collection, formulation of database, data analysis and interpretation, writing the paper.

**Steven Mcguigan, Jonathan Banting:** data collection, data interpretation, writing the paper.

**Shi Hong Shen, Bernhard Riedel:** recruitment of participants, trial governance data collection, data interpretation, writing the paper.

**Mehrdad Nikfarjam, Chris Christophi**: recruitment of participants, data interpretation, writing the paper.

## Conflicts of interest

Edwards Lifesciences supports investigator-initiated research at The Department of Anesthesia Austin Health. A/Prof Laurence Weinberg has received investigator research grants from Edwards Lifesciences. A/Prof Laurence Weinberg is a paid consultant to Edwards Lifesciences. For this study, the conception, design, trial management, data collection, data analyses, and the writing of the manuscript have been executed completely independently of Edwards Lifesciences and any other external organizations.

## Trial registration

Australian New Zealand Clinical Trials Registry number: 12616000172404.

## Guarantor

A/Prof Laurence Weinberg, Director of Anesthesia, Austin Hospital, Heidelberg, 3084,Victoria, Australia; Email: laurence.weinberg@austin.org.au.

Phone: +61 3 94965000.

## Consent

All patients provided their written informed consent for participation in the research study.

## Provenance and peer review

Not commissioned, externally peer reviewed.

## Registration of research studies

International clinical trials registry on 11/02/2016 (Australian New Zealand Clinical Trials Registry number: 12616000172404). There were no changes to the original study protocol endpoints (primary or secondary) approved by Research and Ethics Committee, and participant consent and randomization began after institutional ethics approval was obtained. (Key trial dates: trial protocol finalized: 23/04/2013; submitted for institutional ethics approval: 23/04/13; research ethics committee approval date: 03/09/2013; first participant enrolled: 09/09/2013; last participant enrolled: 23/05/2016; project completion: 01/06/2016).
