# Supplementary material for: Goal directed fluid therapy for major liver resection: A multicentre randomized controlled trial
Source: Ann Med Surg (Lond). 2019 Jul 10;45:45–53. doi: 10.1016/j.amsu.2019.07.003 (PMC6642079; doi:10.1016/j.amsu.2019.07.003)
Supplement: Multimedia component 1 [file mmc1.docx]

**Supplementary Figures**

**Supp. Figure 1:** Changes in mean arterial pressure in patients undergoing liver resection using a physiological cardiac output goal directed therapy (Restrict) (GDT group) algorithm versus Usual care. (Repeated Measures ANOVA p=0.08)

**Supp. Figure 2:** Changes in pulse rate in patients undergoing liver resection using a physiological cardiac output goal directed therapy (Restrict) (GDT group) algorithm versus Usual care. (Repeated Measures ANOVA p=0.88)

**Supp. Figure 3:** Changes in cardiac index in patients undergoing liver resection using a physiological cardiac output goal directed therapy (Restrict) (GDT group) algorithm versus Usual care. (Repeated Measures ANOVA p=0.40)

**Supp. Figure 4:** Changes in stroke volume variation in patients using a physiological cardiac output goal directed therapy (Restrict) (GDT group) algorithm versus Usual care. (Repeated Measures ANOVA p=0.66)

**Supp. Figure 5:** Changes in central venous pressure in patients using a physiological cardiac output goal directed therapy (Restrict) (GDT group) algorithm versus Usual care. (Repeated Measures ANOVA p=0.41)

**Supp. Figure 6:** Changes in stroke volume index in patients using a physiological cardiac output goal directed therapy (Restrict) (GDT group) algorithm versus Usual care. (Repeated Measures ANOVA p=0.83)

**Supp. Figure 7:** Changes in systemic vascular resistance in patients using a physiological cardiac output goal directed therapy (Restrict) (GDT group) algorithm versus Usual care. (Repeated Measures ANOVA p=0.41)
