# Supplementary material for: Goal directed fluid therapy for major liver resection: A multicentre randomized controlled trial
Source: Ann Med Surg (Lond). 2019 Jul 10;45:45–53. doi: 10.1016/j.amsu.2019.07.003 (PMC6642079; doi:10.1016/j.amsu.2019.07.003)
Supplement: Multimedia component 2 [file mmc2.docx]

**Supplementary Table 1:** Overview of the perioperative enhanced recovery protocol.

| **Preoperative** | - Preoperative multidisciplinary evaluation  - Optimization of preoperative comorbidities  - Education of patients and families and informed consent  - Routine preoperative biochemistry, hematology, coagulation, CXR, ECG |
| --- | --- |
| **Anesthesia** | **Day of surgery:** 2 fasting hours for clear fluids, 6 hours for solids  **Anesthesia protocol**  - No preoperative IV fluid loading  **-** Spinal analgesia: intrathecal morphine (200-400ug)  - Induction: propofol (1-mg/kg), fentanyl (3 ug/kg), and non-depolarising neuromuscular blocker  - Maintenance: volatile or propofol infusion (BIS of 40-60)  - Intraoperative analgesia: remifentanil infusion (0.1-0.3 ug/kg/hr)  - Prophylactic thromboembolic prophylaxis (enoxaparin 40mg SC)  - Antibiotic prophylaxis (ceftriaxone/ampicillin/metronidazole)  - Pre-hepatic transection phase: fluid restriction  - Hepatic transection phase: fluid restriction, low central venous pressure using reverse Trendelenburg positioning and glyceryl trinitrate IVI infusion (5-20 ug/min)  - Post hepatic transection phase: restoration of euvolemia  **At completion of surgery**  - Fentanyl infusion 10ug/hr IV  - Ketamine infusion: 0.05-0.1 mg/kg/hr  - Paracetamol 1g IV |
| **Day 0 and 1 postoperatively** | **Analgesia**  - Patient Controlled Analgesia fentanyl: (20ug/bolus, 5 min lockout)  - Fentanyl infusion (10ug/hr) IV  - Ketamine infusion 0.05-0.1 mg/kg//hr IV  - Paracetamol 1 g IV/po TDS  **Fluid intervention**  - Oral fluids encouraged and soft diet  - Balanced crystalloid maintenance therapy: 125ml/hr  - Albumex 4% 250 mls prn at discretion of clinicians  **Other**  - Physiotherapy: early mobilization within 6 hours of surgery  - Metoclopramide 15 mg IVI TDS  - Potassium and magnesium supplementation  - Vitamin K 10 mg daily  - Continue triple antibiotics for 24 hours  - Dihydrogen phosphate ions (14.5 mmol IV TDS)  - Pantroprazole 40 mg IV/po daily  - Heparin 5000IU SC BD |
| **Day 2** | **Analgesia**  - Fentanyl infusion ceased  - PCA fentanyl (20ug/bolus,5 min lockout)  - Ketamine infusion ceased  - Paracetamol 1 g po TDS  - Tramadol 50-100 mg IV/po QID prn  **Fluid intervention**  - Oral fluids encouraged and soft diet  - Maintenance fluid therapy reduced to 83mls/hr  **Other**  - Metoclopramide 15 mg IVI TDS  - Pantroprazole 40 mg po daily  - Vitamin K 10 mg daily  - Dihydrogen phosphate ions (14.5 mmol IV TDS)  - Potassium and magnesium supplementation  - Physiotherapy: early mobilization TDS  - Antithrombotic prophylaxis  - Urinary catheter removed |
| **Day 2** | **Analgesia**  - Continue PCA fentanyl (20ug/bolus,5 min lockout)  - Stop ketamine infusion  - Strict QID paracetamol  - PRN tramadol  **Fluid intervention**  - Aim for neutral fluid balance  - Reduce maintenance fluid therapy to 42 mls/hr  - Soft ward diet  - 4% Albumex 250 mls PRN for filling  **Other**  - Removal of central venous catheter  - Removal of urinary catheter  - Daily weight  - Strict TDS metoclopramide 15 mg IVI  - Potassium and magnesium supplementation  - Pantroprazole 40 mg daily  - Use of diuretic if positive fluid balance (frusemide 10-20mg)  - Strict Metoclopramide 15mg TDS  - Pantroprazole 40 mg daily  - Vitamin K 10 mg daily  - Dihydrogen phosphate ions (14.5 mmol IV TDS)  - Continue antithrombotic prophylaxis  - Physiotherapy: continue mobilization TDS |
| **Day 3 -> discharge** | **Analgesia**  - Fentanyl PCA ceased  - Oxycodone (IR) 10 mg 4 hourly  - Paracetamol 1 g prn TDS  - Tramadol 50-100 mg prn TDS  **Fluid intervention**  - Advance oral diet  **Other**  - Daily weight  - Potassium and magnesium supplementation  - Pantroprazole 40 mg PO  - Continue antithrombotic prophylaxis  - Coloxyl 100mg BD  - Physiotherapy: continue mobilization TDS |
